# Synergistic Toxicity of Combined Exposure to Acrylamide and Polystyrene Nanoplastics on the Gut–Liver Axis in Mice

**DOI:** 10.3390/biology14050523

**Published:** 2025-05-09

**Authors:** Yongchuang Liu, Ruiping Luo, Zhongke Sun, Yidan Zhang, Yuqi Guo, Yanjuan Chen, Lili Li, Zonghao Yue

**Affiliations:** 1 College of Life Sciences and Agronomy, Zhoukou Normal University, Zhoukou 466001, China; 20201064@zknu.edu.cn (Y.L.); ruipingluo@zknu.edu.cn (R.L.); zhangyidan020422@163.com (Y.Z.); guoyuqi772023@163.com (Y.G.); 2 College of Biological Engineering, Henan University of Technology, Zhengzhou 450001, China; sun_microbiol@sina.com; 3 School of Mechanical and Electrical Engineering, Zhoukou Normal University, Zhoukou 466001, China; 20172033@zknu.edu.cn

**Keywords:** acrylamide, nanoplastics, mice, combined toxicity, gut–liver axis

## Abstract

Acrylamide (AA) and nanoplastics (NPs) are common food toxicants. However, their combined toxicity and health risks call for further studies. This study aimed to investigate the combined toxicity of AA and polystyrene NPs (PS-NPs) in mice through drinking water exposure. Co-exposure to AA and PS-NPs aggravated colon and liver damage, including more severe inflammatory infiltration, higher levels of colonic and hepatic pro-inflammatory cytokines, and elevated serum content of lipopolysaccharide and activities of diamine oxidase, alanine aminotransferase, and aspartate aminotransferase compared to single exposures. Co-exposure also significantly downregulated the expression of colonic tight-junction genes ZO-1 and Claudin-5. Metabolomics revealed that co-exposure induced more profound metabolic disorders in the liver, particularly affecting amino acid and carbohydrate metabolism. 16S amplicon sequencing showed that co-exposure caused more drastic gut microbiota dysbiosis, characterized by a decrease in beneficial bacteria (*unclassified_f__Oscillospiraceae*, *Roseburia*, *UCG-005*, *Ruminiclostridium*, *unclassified_o__Clostridia_UCG-014*, *Fournierella*, and *Acetatifactor*) and an increase in pathogenic bacteria (*Eubacterium_xylanophilum_group* and *Eubacterium_nodatum_group*). Correlation analysis indicated a negative correlation between beneficial bacteria and intestinal-liver toxicity indicators and a positive correlation between pathogenic bacteria and these indicators. Overall, our findings showed that AA and PS-NPs exerted synergistic toxicity to the gut–liver axis in mammals, highlighting the higher health risks of their combined ingestion.

## Simple Summary

Acrylamide (AA) and nanoplastics (NPs) are two common food contaminants and have been proven to exhibit multiple toxic effects. They have a high probability of coexisting due to the similar sources. Thus, our study aims to investigate the combined toxicity of AA and NPs on mammals from the perspective of the gut–liver axis. By exposing mice to AA, polystyrene NPs (PS-NPs), and AA + PS-NPs for 10 weeks, we observed that combined exposure to AA and PS-NPs resulted in more significant adverse effects than single exposures, including more severe colon and liver damage, liver metabolic disorders, and gut microbiota dysbiosis. Correlation analysis showed that the synergistic toxicity induced by AA + PS-NPs may be attributed to a decrease in short-chain fatty acid (SCFA)-producing bacteria and an increase in pathogenic bacteria. These findings provide novel insights into the understanding of the combined exposure to these two contaminants, serving as a reference for future research assessing the health risks associated with their simultaneous intake.

## 1. Introduction

During food thermal processing, some hazardous substances are spontaneously generated, of which acrylamide (AA) is the most representative. AA is prevalent in foods, particularly potato chips, bakery products, coffee, and others [1]. In certain items, AA content exceeds 6 mg/kg, well above the European Union standard (Commission Regulation (EU) 2017/2158 [2]) [3]. Moreover, AA can be detected in drinking water and cigarette smoke [4,5]. Epidemiological evidence shows that AA intake is closely associated with cancer, type 2 diabetes, cardiovascular disease, and depressive symptoms [6,7,8,9]. Toxicological studies have indicated that AA induces carcinogenicity, genotoxicity, neurotoxicity, hepatotoxicity, cardiovascular toxicity, and reproductive toxicity [10]. Our previous studies also showed that AA caused glucose metabolism disorders and intestinal damage [11,12,13]. Significant efforts have been made to reduce AA levels in food products [14]. However, preventing AA formation completely is challenging owing to the inherent presence of reducing sugars and asparagine in food [3].

In addition to AA, some exogenous contaminants have been found in foods, such as microplastics (MPs) and nanoplastics (NPs). Numerous studies show that MPs/NPs can be detected in sea salt, aquatic products, honey, fruits, vegetables, and bottled water [15]. Several recent studies have reported that MPs/NPs can be released from plastic disposable cups, food containers, food pouches, and food packaging materials [16,17,18]. Another study also provides evidence of atmospheric MPs/NPs [19]. These findings highlight the pervasiveness of MPs/NPs in the environment and imply that human exposure to these is inevitable. The presence of MPs/NPs has been increasingly confirmed in human tissues, including blood, breast milk, semen, feces, and placenta [20]. Toxicological studies indicate that MPs/NPs can trigger oxidative stress, inflammation, immunosuppression, neurological dysfunction, gut microbiota imbalance, and metabolic disorders [21]. A recent epidemiological investigation reveals a positive correlation between MPs and inflammatory bowel disease severity [22]. Therefore, the potential health risks posed by MPs/NPs to humans cannot be ignored, especially NPs, because of their larger surface area, smaller size, and higher biological permeability, potentially causing more severe toxic effects than those of MPs [23].

As MPs/NPs and AA share common sources (food, water, and air), the risk of humans simultaneously ingesting them is significantly increased. The individual toxic effects of AA and MPs/NPs are well studied; however, research on their combined exposure remains lacking. Only one study has reported the effects of combined AA and PS-NP exposure on zebrafish, demonstrating that co-exposure resulted in more pronounced embryonic developmental inhibition and more severe motor behavioral abnormalities compared to AA exposure alone [24]. The gut–liver axis plays a critical role in maintaining host health by protecting host against hazardous substances [25]. An imbalance in its homeostasis can facilitate the movement of metabolites and gut flora components to the liver, thus leading to liver inflammation and damage [26]. Many toxic contaminants, including AA and MPs/NPs, have been reported to adversely affect the gut–liver axis [12,27]. Therefore, this study aimed to investigate the combined toxicity and underlying mechanism of AA and polystyrene NP (PS-NP) exposure in mice from the perspective of the gut–liver axis through microbial diversity assessment, metabolomics analysis, histopathological observations, and biochemical analysis. Our findings could elucidate the combined toxic effects of AA and NPs on mammals, providing a novel theoretical basis for evaluating potential health risks associated with their combined toxicity.

## 2. Materials and Methods

### 2.1. Materials and Reagents

AA (purity ≥ 99.9%, Cas No. 79-06-1) was purchased from Macklin (Shanghai, China), and 50 nm polystyrene NPs suspension (PS-NPs, 50 mg/mL in ultrapure water) was acquired from Zhongkeleiming (Beijing, China). Scanning electron microscopy (SEM) and Zeta potential analysis revealed that the PS-NPs exhibited a spherical morphology with an average diameter of 78.9 ± 8.6 nm and a surface charge of −15.9 mV (Figure S1). SPF-grade female C57BL/6N mice (6 weeks old) were purchased from Vital River (Beijing, China). Commercial kits for alanine aminotransferase (ALT) and aspartate aminotransferase (AST) were obtained from Boxbio (Beijing, China). ELISA kits for diamine oxidase (DAO), tumor necrosis factor-α (TNF-α), interleukin-1β (IL-1β), IL-6, and IL-17A were purchased from Elabscience (Wuhan, China). Another ELISA kit for lipopolysaccharide (LPS) was obtained from CUSABIO (Wuhan, China).

### 2.2. Animal Experimental Design

Female mice were acclimated for 1 week in a standard animal facility (25 °C and 50% relative humidity). The reason for selecting females in this study is based on a previous report which indicates that females may be more prone to absorb AA than males [28]. The mice were randomly divided into four groups, control, AA exposure (AA), PS-NP exposure (PS-NP), and AA + PS-NP co-exposure (AA + PS-NP) groups, with six mice per group. Mice in the AA and PS-NP groups were administered drinking water containing 20 μg/mL AA and 10 μg/mL PS-NPs, respectively. The AA + PS-NP group drank water containing AA and PS-NPs, while the control group drank water without these additives. AA was dissolved in distilled water to achieve a final concentration of 20 μg/mL. The PS-NP suspension was added to distilled water or 20 μg/mL AA solution to achieve a final concentration of 10 μg/mL. These solutions were sterilized via 0.22 μm membrane filtration prior to animal administration and did not contain any endotoxin. Water consumption was recorded every 5–6 days. All animals were fed an SPF-grade standard diet from KeAo Xieli Feed Co., Ltd. (Beijing, China) with composition details in Supplementary Table S1. Based on water consumption (Table S2), the daily intake of AA per mouse was approximately equivalent to 4.0 mg/kg·bw/day for the AA group and the AA + PS-NP group. The daily intake of PS-NPs per mouse was approximately equivalent to 2.0 mg/kg·bw/day for the PS-NPs group and the AA + PS-NPs group. The selection of AA concentration was based on the high exposure population of AA and our previous study [13], while the selection of PS-NP concentration was based on a previous study, which reported that humans may consume 0.1–5 g of MPs/NPs weekly, equivalent to a daily intake of 0.24–11.9 mg/kg·bw [29]. Detailed reasons for dosage selection have been provided in the Supplementary Methods.

After 10 weeks, all mice were fasted for 8 h and euthanized via intraperitoneal injection of tribromoethanol (350 mg/kg) (Cas No. 75-80-9, Sigma-Aldrich, Saint Louis, MO, USA) following the AVMA Guidelines for the Euthanasia of Animals. Blood was collected via eyeball extirpation, a common method for collecting blood from rodents [30,31], and centrifuged at 800× *g* for 10 min at 4 °C to obtain serum. Serum, cecal contents, and a portion of liver and colon samples were frozen in liquid nitrogen and stored at −80 °C for subsequent analysis. The remaining liver and colon tissues were fixed in 4.0% paraformaldehyde for histopathological analysis.

### 2.3. Biochemical Analyses of Serum, Liver, and Colon

Serum levels of liver injury markers (ALT and AST activities) and intestinal damage markers (DAO activity and LPS content) were measured following the manufacturer′s procedures. The liver and colon tissues were homogenized with PBS (0.01 M, pH 7.4) containing proteinase inhibitor and centrifuged for 10 min at 4 °C, 8000× *g*. The levels of inflammatory markers TNF-α, IL-1β, IL-6, and IL-17A in these homogenates were determined using ELISA kits. The absorbance was measured using a SpectraMax i3x microplate reader (Molecular Devices, San Jose, CA, USA).

### 2.4. Histopathological Analyses of the Liver and Colon

After 24 h of fixation, the liver and colon tissues were embedded in paraffin and sectioned into 5 μm thick slices. Liver sections were stained with hematoxylin and eosin (H&E), while colon sections were stained with H&E or Alcian blue and periodic acid–Schiff (AB-PAS). Histopathological observations were performed using a microscope slide scanner (3DHISTECH, Budapest, Hungary).

### 2.5. Real-Time Quantitative PCR (RT-qPCR) Analysis

Total RNA from the liver and colon samples was extracted, assessed, and reverse-transcribed into cDNA, as previously described [12]. RT-qPCR was performed using the ChamQ SYBR qPCR Master Mix kit (Vazyme, Nanjing, China) in a CFX96 RT-PCR System (BioRad, Hercules, CA, USA). Glyceraldehyde-3-phosphate dehydrogenase (GAPDH) served as the housekeeping gene, and the relative target gene mRNA levels were calculated using the 2^−ΔΔCt^ method. Supplementary Table S3 lists the primer sequences.

### 2.6. Untargeted Metabolomics of Liver and Bioinformatics Analysis

Liver samples were extracted as previously described [12]. The resulting supernatants were analyzed using ultrahigh-performance liquid chromatography tandem mass spectrometry (UPLC-MS/MS). Details on the UPLC-MS/MS analysis are provided in the Supplementary Methods. Raw data from UPLC-MS/MS were processed into a final data matrix for subsequent analyses, with detailed procedures provided in the Supplementary Methods. Principal component analysis (PCA) and orthogonal projections to latent structure discriminant analysis (OPLS-DA) were performed using the R package ropls. Differential metabolites (DMs) between the control and different exposure groups were identified based on the criteria of variable importance in the projection (VIP) > 1.0 and *p* < 0.05. Pathway topology analysis of DMs was conducted using the Kyoto Encyclopedia of Genes and Genomes (KEGG) database, with key metabolic pathways identified at a critical value threshold of ≥0.10.

### 2.7. 16S rRNA Amplicon Sequencing of Cecal Contents and Bioinformatics Analysis

Total DNA was extracted using the HiPure Stool DNA Mini Kit B (Magen Biotech, Guangzhou, China). The V3-V4 region of the 16S rRNA gene was amplified using universal primers 338F (5′-ACTCCTACGGGAGGCAGCAG-3′) and 806R (5′-GGACTACHVGGGTWTCTAAT-3′). PCR products were quantified using QuantiFluor™-ST (Promega, Madison, WI, USA). The sequencing library was constructed and sequenced on the NovaSeq 6000 System (Illumina, San Diego, CA, USA) by Origingene (Shanghai, China). The raw sequencing reads were quality filtered using fastp, merged with FLASH, and denoised using the DADA2 plugin in Qiime2 (v2022.2) to generate a bacterial amplicon sequence variants (ASVs) table. ASV taxonomic assignment was conducted using the Qiime2 and SILVA 16S rRNA database. Based on the ASV data, alpha diversity was analyzed using Mothur (v2022), and beta diversity was analyzed using the R package vegan (v 3.3.1). Additionally, linear discriminant analysis effect size (LEfSe) was used to identify significantly different taxa (phylum to genera) between groups (LDA score > 2.5, *p* < 0.05). All bioinformatic analyses were performed using the Majorbio cloud platform (https://cloud.majorbio.com, accessed on 15 July 2024).

### 2.8. Statistical Analysis

Data are presented as means ± standard deviation (SD) and were analyzed using SPSS Statistics 19.0 (IBM, Armonk, NY, USA). Normality tests (Kolmogorov–Smirnov) and equal variance tests (Levene median) were conducted prior to testing for differences between groups. All data followed a normal distribution. One-way ANOVA, followed by Tukey’s test (for equal variances) or Tamhane’s T2 test (for unequal variances), was used to assess significant differences between the control and different exposure groups. Correlations between the microbiota and intestinal–liver damage indicators were analyzed using Spearman′s correlation analysis. *p* < 0.05 was considered statistically significant.

## 3. Results

### 3.1. Co-Exposure to AA and PS-NPs Aggravates Colonic Inflammation in Mice

**Figure 1:**
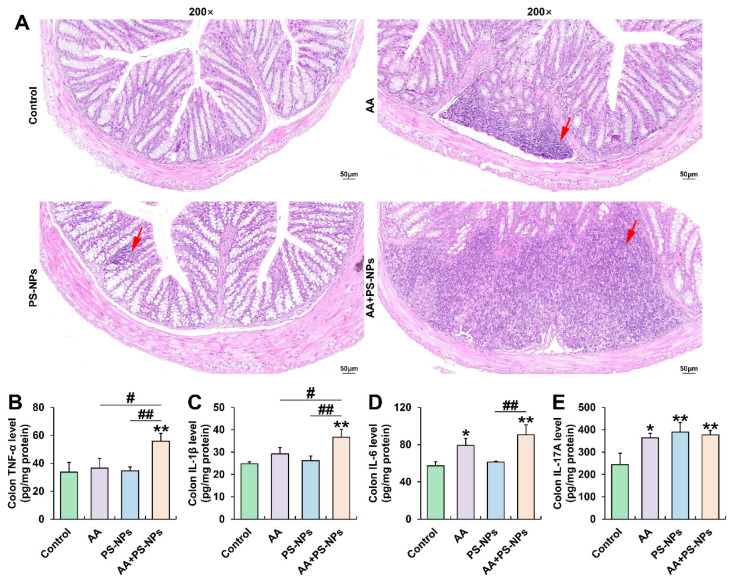
Effects of exposure to AA and/or PS-NPs on colonic histopathology and inflammatory cytokine levels. (**A**) Representative images of colon sections stained with H&E (200×). The red arrows display inflammatory infiltration. (**B**–**E**) Colonic TNF-α, IL-1β, IL-6, and IL-17A levels. Data are presented as means ± SD (n = 3). * *p* < 0.05 and ** *p* < 0.01 compared to the control group; # *p* < 0.05 and ## *p* < 0.01 compared to the AA + PS-NP group.

Exposure to AA and PS-NPs individually resulted in moderate inflammatory infiltration in the colon compared with that in the control group. However, combined exposure to AA and PS-NPs caused severe colonic inflammatory infiltration (Figure 1A). Furthermore, AA exposure significantly increased colonic IL-6 and IL-17A levels, whereas PS-NP exposure significantly increased only IL-17A levels compared with that of the control group (Figure 1D,E). Co-exposure to AA and PS-NPs increased TNF-α, IL-1β, IL-6, and IL-17A levels, with TNF-α and IL-1β levels being higher than those observed under the two single exposure conditions and IL-6 levels being higher than those after PS-NP exposure alone (Figure 1B–D).

### 3.2. Co-Exposure to AA and PS-NPs Damages Colonic Barrier Function in Mice

**Figure 2:**
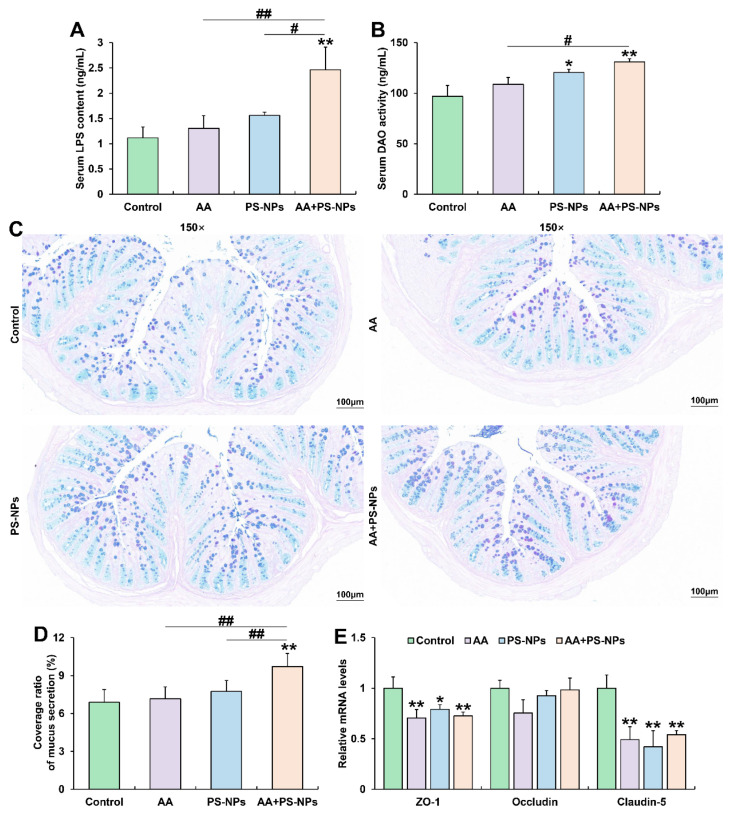
Effects of exposure to AA and/or PS-NPs on mucus secretion and intestinal barrier in colon of mice. (**A**) Serum LPS content. (**B**) Serum DAO activity. (**C**) Representative images of colon sections stained with AB-PAS (150×). (**D**) Coverage ratio of mucus secretion. (**E**) Relative mRNA levels of colonic ZO-1, Occludin, and Claudin-5. Data are presented as means ± SD (n = 3). * *p* < 0.05 and ** *p* < 0.01 compared to control group; # *p* < 0.05 and ## *p* < 0.01 compared to AA + PS-NP group.

Serum LPS content showed an increasing trend with AA or PS-NP exposure alone and significantly increased under AA + PS-NP co-exposure compared to those in the control group (Figure 2A). Similarly, serum DAO activity exhibited a rising trend under AA exposure alone and significantly increased after PS-NP exposure and AA + PS-NP co-exposure compared to that in the control group (Figure 2B). Combined exposure resulted in a higher serum LPS content than that after the two single exposure conditions and a higher serum DAO activity than that after AA exposure alone (Figure 2A,B).

Colonic AB-PAS staining showed no significant differences in the coverage ratio of mucus secretion between the control, AA, and PS-NPs groups. However, co-exposure to AA and PS-NPs significantly increased mucus coverage compared with that in the control, AA and PS-NPs groups (Figure 2C,D). Furthermore, AA, PS-NP, and AA + PS-NP exposures significantly downregulated colonic tight-junction (TJ) protein ZO-1 and Claudin-5 mRNA levels compared with those in the control group (Figure 2E).

### 3.3. Co-Exposure to AA and PS-NPs Exacerbates Liver Injury and Inflammation in Mice

**Figure 3:**
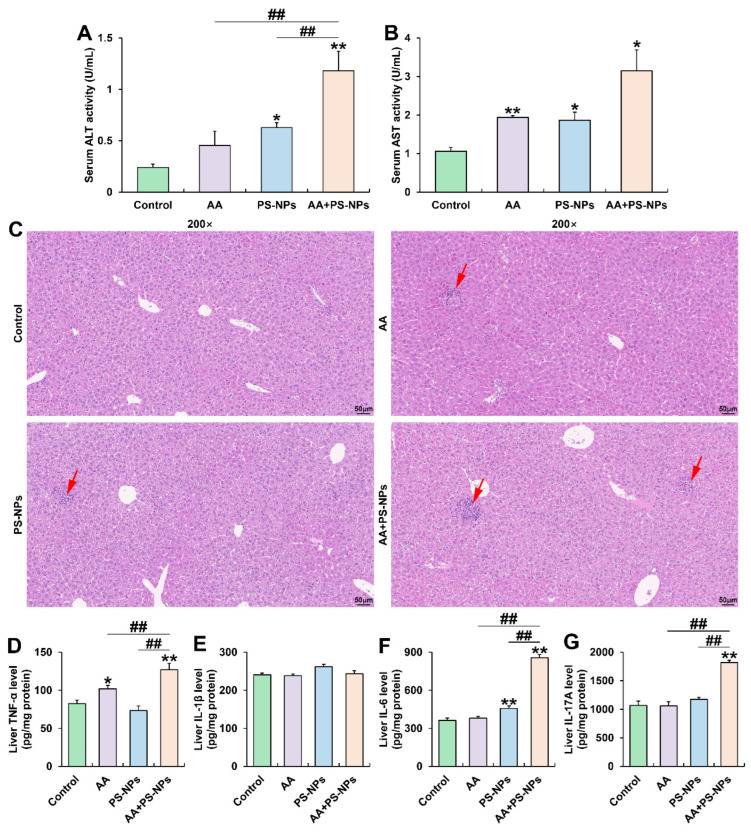
Effects of exposure to AA and/or PS-NPs on serum biomarkers, histopathology, and inflammatory cytokine levels in the liver. (**A**) Serum ALT activity. (**B**) Serum AST activity. (**C**) Representative images of liver sections stained with H&E (200×). The red arrows display inflammatory infiltration. (**D**–**G**) Hepatic TNF-α, IL-1β, IL-6, and IL-17A levels. Data are presented as means ± SD (n = 3). * *p* < 0.05 and ** *p* < 0.01 compared to the control group; ## *p* < 0.01 compared to the AA + PS-NP group.

PS-NP and AA + PS-NP exposures significantly elevated serum ALT and AST activities compared with those in the control group, while AA exposure significantly elevated only ALT activity (Figure 3A,B). Notably, co-exposure to AA + PS-NPs led to higher ALT activity than that due to AA or PS-NP exposure alone (Figure 3A). Histopathological analysis of the liver revealed mild inflammatory infiltration under AA or PS-NP exposure and more obvious cell inflammation under AA + PS-NP co-exposure (Figure 3C). Furthermore, AA exposure significantly elevated TNF-α levels, while PS-NP exposure significantly elevated IL-6 levels compared with those in the control group (Figure 3D,F). Co-exposure to AA and PS-NPs specifically resulted in higher levels of multiple inflammatory cytokines, including TNF-α, IL-6, and IL-17A, than those observed under single exposure conditions (Figure 3D,F,G).

### 3.4. Co-Exposure to AA and PS-NPs Alters Liver Metabolism in Mice

**Figure 4:**
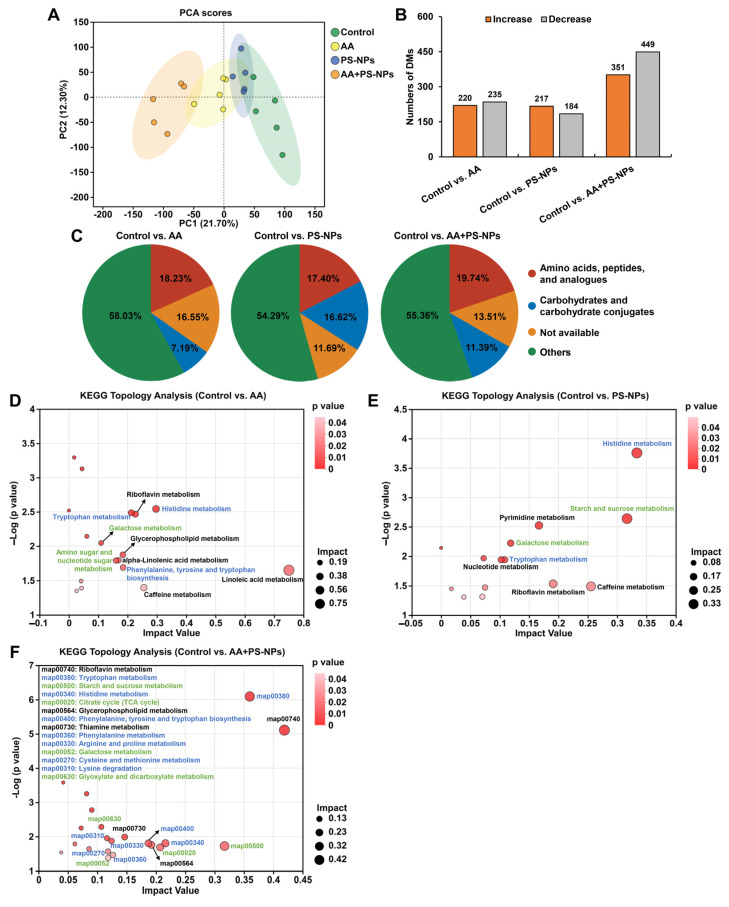
Effects of exposure to AA and/or PS-NPs on liver metabolomics in mice. (**A**) PCA score plot of metabolites obtained by positive and negative ion modes. (**B**) Statistical graph of DMs under AA, PS-NP, and AA + PS-NP exposure compared with those in the control group (n = 5). (**C**) HMDB compound classification of DMs. (**D**–**F**) KEGG topology analysis of DMs. The size and color of each dot represent the pathway impact value and *p* value, respectively. The blue font represents the amino acid metabolism pathways, while the green font represents the carbohydrate metabolism pathways.

PCA results showed a distinct metabolic shift in the AA, PS-NP, and AA + PS-NP groups compared with the control group, with the AA + PS-NP group exhibiting the largest separation, indicating a strong alteration in the liver metabolism of mice (Figure 4A). Using VIP > 1.0 and *p* < 0.05 as the criteria, 455, 401, and 800 DMs were identified in the comparisons of control vs. AA, control vs. PS-NPs, and control vs. AA + PS-NPs, respectively (Figure 4B). According to the Human Metabolome Database (HMDB) compound classification, “amino acids, peptides, and analogues” and “carbohydrates and carbohydrate conjugates” were the most prevalent DMs across all exposure groups (Figure 4C).

KEGG topology analysis revealed that DMs in the AA, PS-NP, and AA + PS-NP groups were significantly enriched in 10, 8, and 14 pathways, respectively (impact value ≥ 0.10, *p* < 0.05; Figure 4D–F). Many of these pathways are involved in amino acid and carbohydrate metabolism. In amino acid metabolism, tryptophan (Trp) metabolism and histidine (His) metabolism were common pathways observed in all exposure groups. Moreover, AA exposure significantly affected phenylalanine, tyrosine, and tryptophan biosynthesis, whereas AA + PS-NP co-exposure had a marked effect on this pathway and significantly disrupted phenylalanine metabolism, arginine and proline metabolism, cysteine and methionine metabolism, and lysine degradation (Figure 4D,F). Among these pathways, kynurenine (KYN), anthranilic acid (ATA), and kynurenic acid (KYNA) contents significantly decreased, while L-histidine content increased across all exposure groups (Table 1). Histamine and urocanic acid contents significantly increased with AA and PS-NP exposures, whereas serotonin (5-HT) increased with AA and AA + PS-NP exposures (Table 1). Furthermore, co-exposure to AA and PS-NPs significantly increased 5-hydroxytryptophan (5-HTP), indole derivatives (indole-3-acetaldoxime, indole-3-acetamide, and indole-3-acetic acid), ergothioneine, methionine sulfoxide (MetO), and saccharopine contents, and it significantly decreased phenylalanine and proline contents (Table 1).

In carbohydrate metabolism, galactose metabolism was consistently affected across all the exposure groups (Figure 4D–F). AA exposure significantly affected amino sugar and nucleotide sugar metabolism. PS-NP and AA + PS-NP exposures significantly perturbed starch and sucrose metabolism. Furthermore, co-exposure to AA and PS-NPs also significantly disrupted the citrate cycle (TCA cycle), as well as glyoxylate and dicarboxylate metabolism (Figure 4D–F). Among these pathways, significant increases in D-glucose and lactose contents were observed under all exposure conditions (Table 1). AA exposure significantly increased glucosamine, UDP-D-galactose, UDP-glucose, and UDP-N-acetylglucosamine contents. PS-NP exposure and AA + PS-NP co-exposure significantly increased trehalose, stachyose, dextran, levan, and galactinol contents (Table 1). Co-exposure to AA and PS-NPs also significantly decreased citric, isocitric, 2-ketoglutaric, glyceric, and glycolic acid contents (Table 1).

### 3.5. Co-Exposure to AA and PS-NPs Changes Cecal Microbiota in Mice

**Figure 5:**
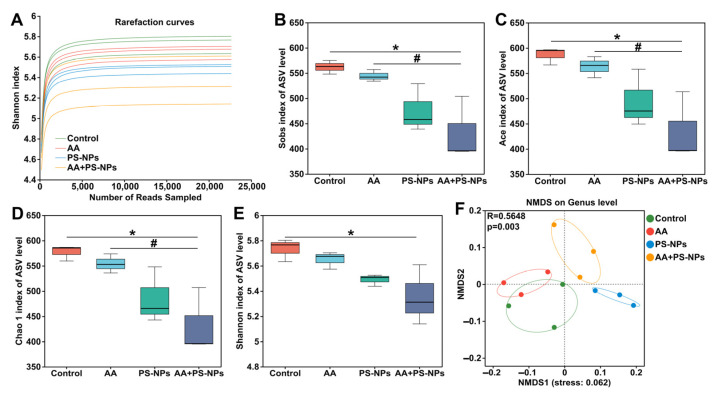
Effects of exposure to AA and/or PS-NPs on microbial diversity of cecal contents of mice. (**A**) The rarefaction curves for the Shannon index at the ASV level for all samples. (**B**–**E**) Microbial alpha diversity analysis: Sobs index (**B**), Ace index (**C**), Chao 1 index (**D**), and Shannon index (**E**). (**F**) NMDS analysis of beta diversity based on the Bray–Curtis distance at the genus level. Data are represented as means ± SD (n = 3). * *p* < 0.05 compared to the control group; # *p* < 0.05 compared to the AA + PS-NP group.

As shown in Figure 5A, the rarefaction curves for the Shannon index of ASVs in each sample gradually plateaued with increasing sequencing depth, indicating sufficient data to accurately capture microbial diversity present in the samples. Alpha diversity analysis revealed no significant differences in richness indices (Sobs, Ace, and Chao 1) or diversity indices (Shannon) between the control group and either the AA or PS-NPs group (Figure 5B–E). However, the AA + PS-NP group showed a significant reduction in these indices compared to the control group (Figure 5B–E). Notably, the Sobs, Ace, and Chao 1 indices observed in AA + PS-NP group were lower than those in AA group. NMDS analysis of beta diversity revealed a distinct separation of all exposure groups from the control group at the genus level (ANOSIM R = 0.5648, *p* = 0.003) (Figure 5F).

**Figure 6:**
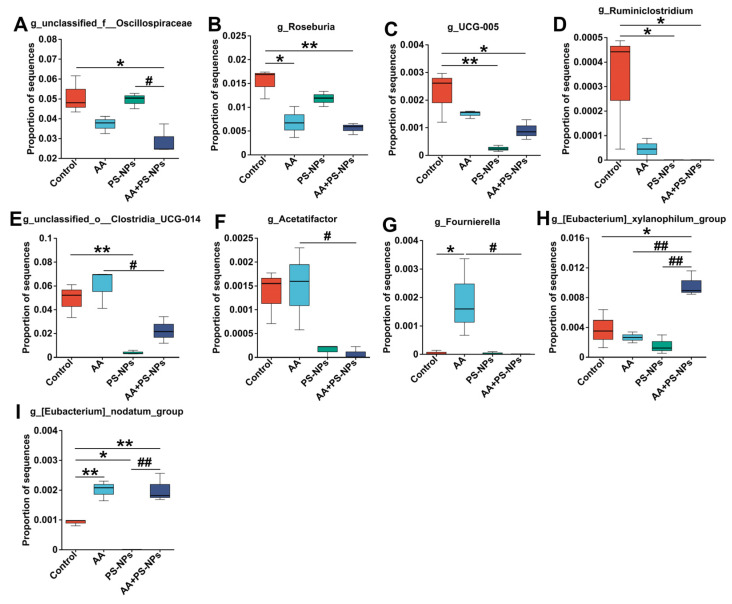
Differentially abundant microbes at the genus level between groups. (**A**) *unclassified_f__Oscillospiraceae*. (**B**) *Roseburia*. (**C**) *UCG-005*. (**D**) *Ruminiclostridium*. (**E**) *unclassified_o__Clostridia_UCG-014*. (**F**) *Acetatifactor*. (**G**) *Fournierella*. (**H**) *Eubacterium_xylanophilum_group*. (**I**) *Eubacterium_nodatum_group*. Data are represented as means ± SD (n = 3). * *p* < 0.05 and ** *p* < 0.01 compared to the control group; # *p* < 0.05 and ## *p* < 0.01 compared to the AA + PS-NP group.

To further identify differentially abundant microbes among the different exposure groups, LEfSe was used, revealing 35 bacterial taxa with differential abundance (LDA > 2.5, *p* < 0.05) (Figures S2 and S3). Compared with those in the control group, *Roseburia* abundance significantly decreased, whereas *Fournierella* and *Eubacterium_nodatum_group* abundances significantly increased in the AA group (Figure 6B,G,I). PS-NP exposure resulted in a significant decrease in the abundances of *UCG-005*, *Ruminiclostridium*, *unclassified_o__Clostridia_UCG-014*, and *Eubacterium_nodatum_group* (Figure 6C–E,I). In contrast, AA + PS-NP co-exposure significantly decreased the abundances of *unclassified_f__Oscillospiraceae*, *Roseburia*, *UCG-005*, and *Ruminiclostridium*, accompanied by a significant increase in *Eubacterium_xylanophilum_group* and *Eubacterium_nodatum_group* (Figure 6A–D,H,I). Compared to the AA group, AA + PS-NP co-exposure significantly reduced the abundances of *unclassified_o__Clostridia_UCG-014*, *Acetatifactor*, and *Fournierella*, whereas increasing that of *Eubacterium_xylanophilum_group* (Figure 6E–H).

### 3.6. Correlation Analysis Between Gut Microbiota and Related Intestinal–Liver Indicators

**Figure 7:**
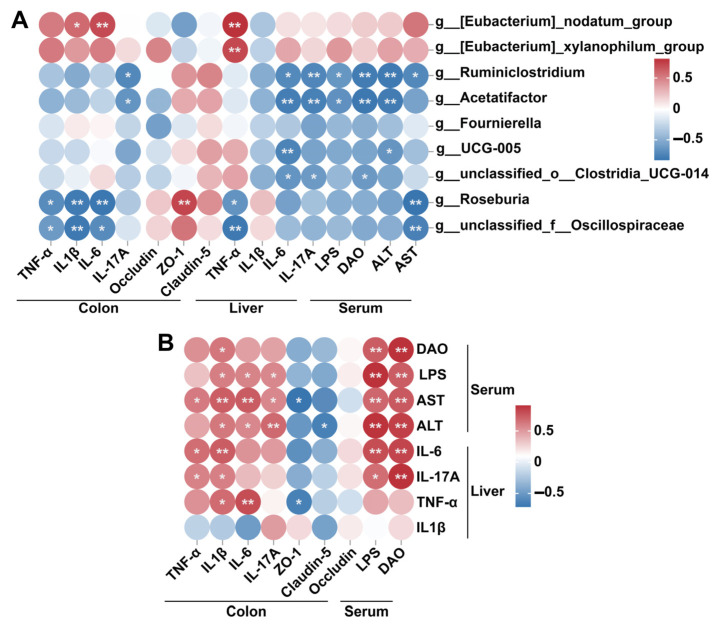
Spearman′s correlation analysis between differentially abundant genera and related indicators involved in intestinal damage and liver injury (**A**), and between intestinal damage-related indicators and liver injury related indicators (**B**). * *p* < 0.05; ** *p* < 0.01.

Spearman′s correlation analysis revealed that *Eubacterium_nodatum_group* was positively correlated with colonic IL-1β and IL-6 and liver TNF-α, whereas *Eubacterium_xylanophilum_group* was positively correlated with liver TNF-α (Figure 7A). In contrast, *unclassified_f__Oscillospiraceae* and *Roseburia* were negatively correlated with serum AST, liver TNF-α, and colonic TNF-α, IL-1β, and IL-6. *Ruminiclostridium* and *Acetatifactor* were negatively correlated with serum ALT, LPS, DAO, liver IL-6, IL-17A, and colonic IL-17A. Furthermore, *UCG-005* was negatively correlated with serum ALT and liver IL-6, whereas *unclassified_o__Clostridia_UCG-014* was negatively correlated with serum DAO, and liver IL-6, IL-17A. Colonic ZO-1 was positively correlated with *Roseburia*. Additionally, serum ALT and AST were positively correlated with serum LPS, DAO, and colonic IL-6, IL-17A, and IL-1β (Figure 7B). Conversely, ALT and AST were negatively correlated with colonic ZO-1 and Claudin-5, respectively. Serum LPS were positively correlated with colonic IL-6, IL-17A, and IL-1β, while serum DAO is only positively correlated with colonic IL-1β (Figure 7B). Liver IL-6 and IL-17A were positively correlated with serum LPS, DAO, and colonic TNF-α, IL-1β. Liver TNF-α was positively correlated with colonic IL-1β and IL-6 and negatively correlated with colonic ZO-1 (Figure 7B).

## 4. Discussion

Evidence indicates that AA and NPs can have various toxic effects, including intestinal and liver damage [10,32]. Several studies have shown the combined effects of AA or NPs with other pollutants [33,34,35,36]. However, the toxicological data on combined exposure to AA and NPs remains limited. The findings of this study revealed that co-exposure to AA and PS-NPs produced synergistic toxicity and aggravated gut–liver axis impairment in mice, including colonic inflammation, intestinal barrier damage, hepatic inflammation, liver metabolism remodeling, and gut microbiota dysbiosis.

Previous studies show that exposure to AA or NPs alone can cause colonic inflammation and increase pro-inflammatory cytokine levels [37,38]. Similar results were also observed in this study. Co-exposure to AA and PS-NPs resulted in more severe inflammatory infiltration and higher pro-inflammatory cytokine levels (TNF-α, IL-1β, and IL-6) than those due to AA or PS-NP exposure alone. Consistent with our findings, combined exposure to PS-NPs with aflatoxin B1 or cadmium exhibited synergistic effects on colonic inflammation in mice [35,36].

Intestinal inflammation is closely associated with intestinal barrier dysfunction. An intact intestinal barrier is essential for maintaining intestinal homeostasis and preventing the entry of harmful substances and pathogens [39]. The mucus layer serves as the first line of defense, crucial for preventing bacterial invasion and inflammation in the intestinal epithelium [40]. Here, AB-PAS staining showed that co-exposure to AA and PS-NPs resulted in a higher coverage ratio of mucus secretion than that in the control, AA, and PS-NP groups. When severe intestinal inflammation occurs, intestinal nociceptor neurons prompt goblet cells to produce mucus, thereby protecting the gut barrier [41]. This increased colonic mucus secretion may serve as a self-protective reaction to cope with the severe colonic inflammation induced by combined exposure. Furthermore, we observed higher serum LPS content and DAO activity after co-exposure to AA and PS-NPs. Serum LPS content and DAO activity are established biomarkers for assessing intestinal mucosal damage and barrier permeability [42]. Therefore, these results indicated that co-exposure caused more severe intestinal damage. Spearman′s correlation analysis showed that the increase of these two markers is positively correlated with the elevated levels of intestinal inflammatory factors. Additionally, all exposure groups significantly downregulated colonic TJ protein ZO-1 and Claudin-5 mRNA levels, compared with the control group. These TJ proteins are crucial for maintaining the integrity and permeability of the intestinal barrier [43]. Therefore, their downregulation may further exacerbate intestinal barrier damage.

Serum LPS enters the liver via the portal vein, triggering inflammatory reactions that subsequently lead to liver injury [44,45]. In mammals, serum ALT and AST are sensitive biomarkers of liver injury [46]. Here, co-exposure to AA and PS-NPs resulted in higher serum AST and ALT activities, more severe liver inflammatory infiltration, and higher levels of hepatic inflammatory cytokines (TNF-α, IL-6, and IL-17A) compared to exposure to AA or PS-NPs alone, indicating that combined exposure induces more severe liver injury and inflammation. The correlation analysis further revealed that the increase in these liver injury markers was strongly positively correlation with serum LPS content.

Tissue injury is often accompanied by changes in endogenous metabolites [47,48]. Hence, we investigated the effects of AA and/or PS-NP exposure on liver metabolism. The results showed that the AA + PS-NP co-exposure group produced more DMs compared with those in the AA and PS-NP exposure groups, indicating that combined exposure exacerbates metabolic disorders in the liver. Pathway enrichment analysis revealed that amino acid and carbohydrate metabolism were key pathways affected by AA, PS-NP, and AA + PS-NP exposures. In amino acid metabolism, all three exposure conditions perturbed Trp and His metabolism. As an essential amino acid, 90–95% of Trp is catabolized into bioactive substances via the KYN pathway in the liver, while 1–2% of Trp is converted into 5-HTP and 5-HT via the 5-HT pathway in enterochromaffin cells, and an additional 5% is transformed into indoles and its derivatives via the indole pathway mediated by gut microbiota [49]. Our findings showed that exposure to AA, PS-NPs, and AA + PS-NPs significantly reduced KYN, ATA, and KYNA contents, suggesting the inhibition of the KYN pathway. Moreover, AA exposure significantly increased 5-HT, and AA + PS-NP co-exposure significantly increased 5-HTP, 5-HT, and indole derivatives. 5-HTP, 5-HT, and indole derivatives are intestinal metabolites of Trp. The increased translocation of these intestinal metabolites to the liver reflected an increase in intestinal permeability. His is another essential amino acid for organisms that can be converted into histamine by histidine decarboxylase or ergothioneine by cyanobacteria, mycobacteria, and fungi [50]. Here, AA and PS-NP exposure significantly increased histamine contents, whereas AA + PS-NP co-exposure significantly increased ergothioneine content. Histamine is a crucial inflammatory mediator and can stimulate and intensify the inflammatory response [51]. Therefore, the increase in histamine induced by AA and PS-NPs may partly explain its role in liver inflammation. The increased ergothioneine in the liver further supported the potential role of AA + PS-NP co-exposure in causing intestinal barrier damage. In addition to the amino acid pathways described above, co-exposure to AA + PS-NPs also disrupted other amino acid pathways. In particular, combined exposure significantly decreased phenylalanine and proline contents, while significantly increasing MetO and saccharopine contents. Phenylalanine is an indispensable precursor for other amino acids and catecholamine neurotransmitter biosynthesis [52]. Therefore, a reduction in phenylalanine content may affect the production of these neurotransmitters, resulting in neurological disorders. Proline is a nonessential amino acid, and its oral administration effectively suppresses hepatic inflammatory infiltration and reduces serum TNF-α, AST, and ALT levels in rats [53]. Therefore, decreased proline content may be associated with AA + PS-NP-induced liver injury. MetO, a major methionine oxidation product, is a biomarker for oxidative stress [54]. This increase indicated that co-exposure to AA and PS-NPs may cause ROS overactivation. Saccharopine, a lysine degradation intermediate, is a mitochondrial toxin. Its abnormal accumulation can cause mitochondrial damage in mouse liver [55]. Therefore, its increase upon AA + PS-NP co-exposure may disrupt mitochondrial homeostasis.

In carbohydrate metabolism, AA disrupted amino sugar, nucleotide sugar, and galactose metabolism, whereas PS-NPs and AA + PS-NPs affected starch, sucrose, and galactose metabolism. D-glucose and lactose contents significantly increased in all exposure groups compared with the control group, with the highest level observed under AA + PS-NP co-exposure. Consistent with our findings, some studies have shown that AA or PS-NPs can affect glucose metabolism and increase the glucose content in mice [11,13,56]. Moreover, trehalose and galactinol contents significantly increased under PS-NP exposure and AA + PS-NP co-exposure. Trehalose is a protective agent against environmental stresses and exerts hepatoprotective effects by diminishing inflammatory signaling and bolstering antioxidant defense [57]. This increase may reflect a self-protective mechanism to cope with liver damage. Galactitol, a reducing product of galactose, is a hepatotoxin. Its increase typically causes cell hyperosmotic stress, induces oxidative stress, and triggers ROS accumulation, ultimately resulting in liver injury [58,59]. Additionally, co-exposure of AA + PS-NPs disturbed energy metabolism, as evidenced by a decrease in intermediates of the TCA cycle and glyoxylate and dicarboxylate metabolism, including citric, isocitric, 2-ketoglutaric, glyceric, and glycolic acid. The TCA cycle is a central metabolic pathway involving sugars, lipids, and amino acid metabolism, and it is pivotal in cellular energy production/supply [60]. Glyoxylate and dicarboxylate metabolism is closely related to the TCA cycle and contributes to energy metabolism [61]. Therefore, the reduction in these vital metabolites owing to AA + PS-NP co-exposure indicated that the combined exposure inhibited energy metabolism, which may lead to insufficient energy supply.

Accumulating evidence highlights the crucial role of gut microbiota dysbiosis in host intestinal barrier damage, liver injury, and metabolic disorders [25,62,63]. Here, co-exposure to AA and PS-NPs caused a significant reduction in richness and diversity of cecal contents, indicating that combined exposure exacerbated microbial dysbiosis. Specifically, AA + PS-NP co-exposure significantly decreased the abundances of *unclassified_f__Oscillospiraceae*, *Roseburia*, *UCG-005*, and *Ruminiclostridium* and significantly increased the abundances of *Eubacterium_xylanophilum_group* and *Eubacterium_nodatum_group* compared to the control group. Moreover, co-exposure led to lower abundances of *unclassified_o__Clostridia_UCG-014*, *Fournierella*, and *Acetatifactor* than AA exposure. Among these genera, *unclassified_f__Oscillospiraceae*, *Roseburia*, *UCG-005*, *Ruminiclostridium*, and *Acetatifactor* were the main producers of short-chain fatty acids (SCFAs) [64,65,66,67,68]. SCFAs are essential for maintaining intestinal barrier integrity and suppressing inflammation [69]. The bacteria *unclassified_o__Clostridia_UCG-014* and *Fournierella* are generally considered beneficial. *Unclassified_o__Clostridia_UCG-014* has been linked to improvements in conditions including ulcerative colitis, hyperlipidemia, and fatty liver disease, whereas *Fournierella* is closely associated with immune cell development and pathogen elimination in broiler chickens [70,71,72]. Additionally, some studies showed that *Eubacterium_xylanophilum* and *Eubacterium_nodatum_group* may be pathogenic bacteria positively associated with intestinal damage and metabolic disorders [73,74,75]. Correlation analysis revealed a negative correlation between these beneficial bacteria and intestinal–liver toxicity markers, whereas the two pathogenic bacteria exhibited a positive correlation with these markers. Consequently, AA + PS-NP co-exposure may exacerbate intestine and liver injury by significantly decreasing the levels of these beneficial bacteria while increasing the presence of pathogenic bacteria.

## 5. Conclusions

Overall, this study uncovered that co-exposure to AA and PS-NPs caused more severe gut microbiota dysbiosis, consequently aggravating colonic barrier damage, liver injury, and hepatic metabolic perturbation. Our findings emphasize the synergistic toxicity and health risks associated with simultaneous ingestion of AA and PS-NPs, providing a theoretical basis for formulating effective food safety policies. However, we must concede that this study has certain limitations, as it employed standard spherical NPs, whereas non-spherical morphologies may predominate in actual environments. Furthermore, a study has reported that non-spherical NPs cause stronger toxicity in earthworms than their spherical counterparts [76]. In future work, we will further investigate the co-exposure effects of non-spherical and multi-scale NPs with AA to elucidate the morphology- and size-dependent interactions of PS-NPs under combined exposure.

## Figures and Tables

**Table 1:** The representative DMs involved in amino acid and carbohydrate metabolism pathways under AA, PS-NP, and AA + PS-NP exposures.

Metabolite	M/Z	Control vs. AA			Control vs. PS-NPs			Control vs. AA + PS-NPs		
		VIP	FC	Regulate	VIP	FC	Regulate	VIP	FC	Regulate
**Amino acids metabolism**										
Kynurenine	207.079	1.280	0.958	**↓**	1.280	0.958	**↓**	1.310	0.925	**↓**
Anthranilic acid	136.041	1.702	0.936	**↓**	1.702	0.936	**↓**	1.854	0.854	**↓**
Kynurenic acid	190.049	1.886	0.931	**↓**	2.246	0.919	**↓**	1.864	0.879	**↓**
L-Histidine	156.076	1.549	1.036	**↑**	1.549	1.036	**↑**	1.327	1.053	**↑**
Histamine	110.073	1.222	1.051	**↑**	1.222	1.051	**↑**			
Urocanic acid	137.036	1.284	1.046	**↑**	1.284	1.046	**↑**			
Serotonin	177.101	1.526	1.057	**↑**				1.914	1.142	**↑**
5-Hydroxytryptophan	219.079							1.199	1.059	**↑**
Indole-3-acetamide	175.086							1.888	1.106	**↑**
Indole-3-acetaldoxime	175.085							2.405	1.301	**↑**
Indole-3-acetic acid	158.059							2.052	1.142	**↑**
Ergothioneine	230.094							1.638	1.095	**↑**
Methionine sulfoxide	166.052							1.487	1.071	**↑**
Saccharopine	318.163							1.182	1.057	**↑**
Phenylalanine	331.163							1.525	0.911	**↓**
L-proline	116.070							1.017	0.977	**↓**
**Carbohydrate metabolism**										
Lactose	377.087	2.033	1.126	**↑**	2.642	1.175	**↑**	2.279	1.233	**↑**
D-Glucose	225.063	1.050	1.038	**↑**	1.700	1.073	**↑**	1.265	1.081	**↑**
UDP-D-galactose	565.050	1.850	1.080	**↑**						
UDP-glucose	565.050	1.792	1.099	**↑**						
UDP-N-acetylglucosamine	606.077	1.535	1.059	**↑**						
Glucosamine	162.075	1.092	1.024	**↑**						
Stachyose	665.217				4.615	1.640	↑	3.423	1.686	**↑**
Dextran	549.169				4.026	1.335	↑	3.156	1.393	**↑**
Levan	487.163				3.875	1.243	↑	3.075	1.324	**↑**
Trehalose	325.111				3.726	1.199	↑	2.612	1.218	**↑**
Galactinol	387.116				2.110	1.108	↑	1.620	1.118	**↑**
Citric acid	191.021							1.429	0.909	**↓**
Isocitric acid	191.021							1.345	0.924	**↓**
Glyceric acid	105.020							1.297	0.929	**↓**
2-Ketoglutaric acid	145.015							1.139	0.918	**↓**
Glycolic acid	75.009							1.069	0.955	**↓**

Note: ↑: increase, ↓: decrease.

## Data Availability

All data are available in the article and Supplementary Materials. The 16S amplicon sequencing raw data were uploaded to the NCBI Sequence Read Archive (accession number: PRJNA1158308). Data will be made available on request.

## Supplementary Materials

The following supporting information can be downloaded at: https://www.mdpi.com/article/10.3390/biology14050523/s1, Figure S1: Morphological characterization of PS-MPs using scanning electron microscopy (SEM) (30×); Figure S2: The Cladogram bacterial taxa analyzed by LEfSe (LDA score > 2.5, *p* < 0.05); Figure S3: LDA score histogram from the phylum to genus level by LEfSe (LDA score > 2.5, *p* < 0.05); Table S1: The compositions of SPF-grade standard diet; Table S2: Drinking water records (mL/mouse/day); Table S3: The primer sequences for qPCR assay. Refs. [29,77,78,79,80,81,82,83] are cited in Supplementary Materials.
