# Supplementary material for: Synergistic Toxicity of Combined Exposure to Acrylamide and Polystyrene Nanoplastics on the Gut–Liver Axis in Mice
Source: Biology (Basel). 2025 May 9;14(5):523. doi: 10.3390/biology14050523 (PMC12109039; doi:10.3390/biology14050523)
Supplement: Supplementary file 1 [file biology-14-00523-s001.zip › biology-3572896-supplementary.pdf]

Supplementary Materials

Synergistic Toxicity of Combined Exposure to Acrylamide and Polystyrene Nanoplastics on the Gut–Liver Axis in Mice

Yongchuang Liu [1], Ruiping Luo [1], Zhongke Sun [2], Yidan Zhang [1], Yuqi Guo [1], Yanjuan Chen [3], Lili Li [1,*] and Zonghao Yue [1,*]

[1] College of Life Sciences and Agronomy, Zhoukou Normal University, Zhoukou 466001, China; 20201064@zknu.edu.cn (Y.L.); ruipingluo@zknu.edu.cn (R.L.); zhangyidan020422@163.com (Y.Z.); guoyuqi772023@163.com (Y.G.)

[2] College of Biological Engineering, Henan University of Technology, Zhengzhou 450001, China; sun_microbiol@sina.com

[3] School of Mechanical and Electrical Engineering, Zhoukou Normal University, Zhoukou 466001, China; 20172033@zknu.edu.cn

* Correspondence: lilili@zknu.edu.cn (L.L.); yzh@zknu.edu.cn (Z.Y.)

Supplementary Methods

1. Dosing

The Joint FAO/WHO Expert Committee on Food Additives (JECFA) estimated that the average dietary acrylamide (AA) exposure in humans was 1.0–4.0 μg/kg·bw/day [1]. It was reported that the tolerable daily intake (TDI) of AA in humans is 40 μg/kg·bw/day for neurotoxicity and 2.6 μg/kg·bw/day for cancer [2]. In rodents, the no observed adverse effect level (NOAEL) was 200 μg/kg·bw/day for neurotoxicity, and the benchmark dose lower confidence limit for a 10% response (BMDL10) was 300 μg/kg·bw/day for cancer [3]. This indicates that the sensitivity index between humans and mice to AA ranges from 5 to 115.4. Additionally, the dose equivalent index between humans and mice was 12.3 [4]. Therefore, the equivalent dose of AA in mice should range from 0.25–5.68 mg/kg·bw/day based on a human exposure level of 4.0 μg/kg·bw/day. In this study, we chose 20 mg/L as the exposure dose of AA to reflect environmental exposure in a highly exposed population.

For microplastics/nanoplastics (MPs/NPs), a previous study estimated that humans may ingest 0.1–5.0 g of MPs/NPs weekly via various exposure pathways [5]. The human intake is approximately 0.24–11.9 mg/kg·bw/day based on an average adult body weight of 60 kg. Therefore, we exposed mice to 10 mg/L of PS-NPs.

2. UPLC-MS/MS analysis

UPLC-MS/MS analysis was performed using an ExionLC AD UPLC system coupled with an AB SCIEX Triple TOF 5600+ mass spectrometer (AB Sciex, Framingham, USA). The extracted supernatants from liver samples were injected into an ACQUITY UPLC HSS T3 column (100 mm × 2.1 mm i.d., 1.8 μm; Waters, Milford, USA) at 40 °C. The injection volume was 10.0 μL and the flow rate was 0.40 mL/min. The mobile phase A was 95% water + 5% acetonitrile (containing 0.1% formic acid), and mobile phase B was 47.5% acetonitrile + 47.5% isopropanol + 5% water (containing 0.1% formic acid). The eluent conditions were as follows: 0-5% B for 1.5 min, 5-13% B for 1.0 min, 13-30% B for 2.0 min, 30-100% B for 0.5 min and 100% B for 1.3 min; 100-0% B for 0.1 min and 0% B for 1.6 min. The resulting liquid samples were ionized by electrospray ionization (ESI), and the mass spectrometry (MS) spectra were acquired in positive and negative ion scanning modes, respectively. The detailed MS parameters were as follows: scan type (m/z) 50-1000; ion source gas I and II 50 psi; curtain gas 30 psi; source temperature

550 °C; ion spray voltage floating 5000 V (ESI+), -4000 V (ESI-); declustering potential 80 V; collision energy 40 ± 20 eV; cycle time 510 ms.

3. Processing of raw data from UPLC-MS/MS

The raw data from UPLC-MS/MS were imported into Progenesis QI 2.3 software (Waters, Milford, USA), which generated a data matrix that included retention time, mass-to-charge ratio (m/z), and peak intensity. Meanwhile, metabolite identification was conducted using the Human Metabolome Database (HMDB), Metlin, and Majorbio's self-built database, based on MS spectral information. Afterwards, the data matrix was uploaded to the Majorbio cloud platform (https://www.majorbio.com) for preprocessing, which encompassed missing value filtering and imputation, QC validation, data normalization and log transformation. Following this preprocessing, the final data matrix was obtained and utilized for subsequent analysis.

References

1. The Joint FAO/WHO Expert Committee on Food Additives (JECFA). *Evaluation of Certain Food Additives and Contaminants: Seventy-Second Report of the Joint FAO/WHO Expert Committee on Food Additive*; WHO Technical Report Series; WHO: Geneva, Switzerland, 2011; p. 959.
2. Tardiff, R.G.; Gargas, M.L.; Kirman, C.R.; Carson, M.L.; Sweeney, L.M. Estimation of safe dietary intake levels of acrylamide for humans. *Food Chem. Toxicol.* **2010**, *48*, 658–667. https://doi.org/10.1016/j.fct.2009.11.048
3. The Joint FAO/WHO Expert Committee on Food Additives (JECFA). *Evaluation of Certain Food Additives and Contaminants: Sixty-Fourth Report of the Joint FAO/WHO Expert Committee on Food Additive*; WHO Technical Report Series; WHO: Geneva, Switzerland, 2005; p. 930.
4. Reagan-Shaw, S.; Nihal, M.; Ahmad, N. Dose translation from animal to human studies revisited. *FASEB. J.* **2008**, *22*, 659–661. https://doi.org/10.1096/fj.07-9574LSF

5 Senathirajah, K.; Attwood, S.; Bhagwat, G.; Carbery, M.; Wilson, S.; Palanisami, T. Estimation of the mass of microplastics ingested—A pivotal first step towards human health risk assessment. *J. Hazard. Mater.* **2021**, *404*, 124004. https://doi.org/10.1016/j.jhazmat.2020.124004.

Supplementary tables

Table S1 Compositions of SPF-grade standard diet

	Component	Content		Component	Content		Component	Content
Vitamin	Vitamin A (IU/kg)	20000.00	Mineral	Sodium (g/kg)	2.83	Amino acids	Methionine+Cysteine (g/kg)	8.00
	Vitamin D (IU/kg)	1667.00		Magnesium (g/kg)	2.77		Lysine (g/kg)	13.90
	Vitamin E (mg/kg)	182.00		Potassium (g/kg)	8.20		Tryptophan (g/kg)	2.50
	Vitamin K (mg/kg)	8.00		Copper (mg/kg)	12.41		Arginine (g/kg)	12.00
	Vitamin B1 (mg/kg)	20.23		Iron (mg/kg)	158.60		Leucine (g/kg)	17.60
	Vitamin B2 (mg/kg)	20.00		Manganese (mg/kg)	88.10		Isoleucine (g/kg)	10.50
	Vitamin B6 (mg/kg)	15.00		Zinc (mg/kg)	50.70		Threonine (g/kg)	8.80
	Vitamin B12 (mg/kg)	0.03		Selenium (mg/kg)	0.20		Valine (g/kg)	11.90
	Niacin (mg/kg)	70.00		Iodine (mg/kg)	0.90		Histidine (g/kg)	5.60
	Pantothenic acid (mg/kg)	25.00					Phenylalanine+tyrosine (g/kg)	16.80
	Biotin (mg/kg)	0.30						
	Choline (mg/kg)	1250.00						
	Folic acid (mg/kg)	10.00						

Table S2 Drinking water records (mL/mouse/day)

Group	Record													
	1	2	3	4	5	6	7	8	9	10	11	12	Mean	SD
**Control**	5.27	4.61	5.06	5.44	5.11	4.89	4.72	4.64	4.67	4.61	4.50	4.33	4.82	0.33
**AA**	5.00	4.89	4.44	4.78	4.67	4.67	4.50	4.39	4.72	4.50	4.33	4.60	4.62	0.20
**PS-NPs**	5.50	4.69	5.19	4.94	4.89	4.83	4.94	4.72	4.72	4.33	5.00	5.27	4.92	0.30
**AA+PS-NPs**	5.08	4.33	4.56	4.53	5.06	4.78	4.53	4.72	4.72	4.56	4.89	4.80	4.71	0.23

Table S3 Primer sequences for qPCR assay

Gene name	Primer sequence (5′–3′)	Reference
ZO-1	F: GAGTTTGACAGTGGAGTCG	Zhao et al., 2021
	R: AGCTGAAGGACTCACAGGAA	
Claudin-5	F: CTACTAGCTGGGCTACTCACA	
	R: AGCAGGGAGAGGAAACAAAAGT	
Occludin	F: TCGGTACAGCAGCAACGATAA	Liu et al., 2022
	R: CTGTCGTGTAGTCGGTTTCATA	
GAPDH	F: GGTTGTCTCCTGCGACTTCA	Chen et al., 2021
	R: TGGTCCAGGTTTCTTACTCC	

Reference

Chen, S.N.; Tan, Y.; Xiao, X.C.; Li, Q.; Wu, Q.; Peng, Y.Y.; Ren, J.; Dong, M.L. Deletion of TLR4 attenuates lipopolysaccharide-induced acute liver injury by inhibiting inflammation and apoptosis. *Acta Pharmacol. Sin*. **2021**, *42*, 1610–1619.

Liu, X.; Zhang, Y.; Li, W.; Zhang, B.; Yin, J.; Liuqi, S.; Wang, J.; Peng, B.; Wang, S. Fucoidan ameliorated dextran sulfate sodium-induced ulcerative colitis by modulating gut microbiota and bile acid metabolism. *J. Agric. Food Chem.* **2022**, *70*, 14864–14876.

Zhao, Z.; Ning, J.; Bao, X.Q.; Shang, M.; Ma, J.; Li, G.; Zhang, D. Fecal microbiota transplantation protects rotenone-induced Parkinson's disease mice via suppressing inflammation mediated by the lipopolysaccharide-TLR4 signaling pathway through the microbiota-gut-brain axis. *Microbiome* **2021**, *9*, 226.

Supplementary figures

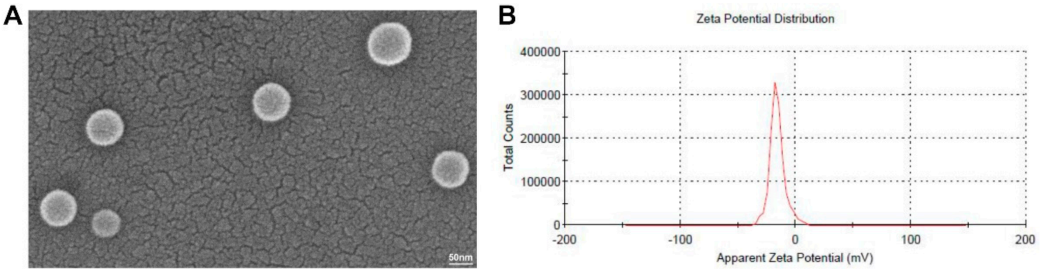

Figure S1 Morphological characterization (A) and Zeta potential analysis of PS-MPs using scanning electron microscopy (SEM) (30×) and zeta potential analyzer, respectively

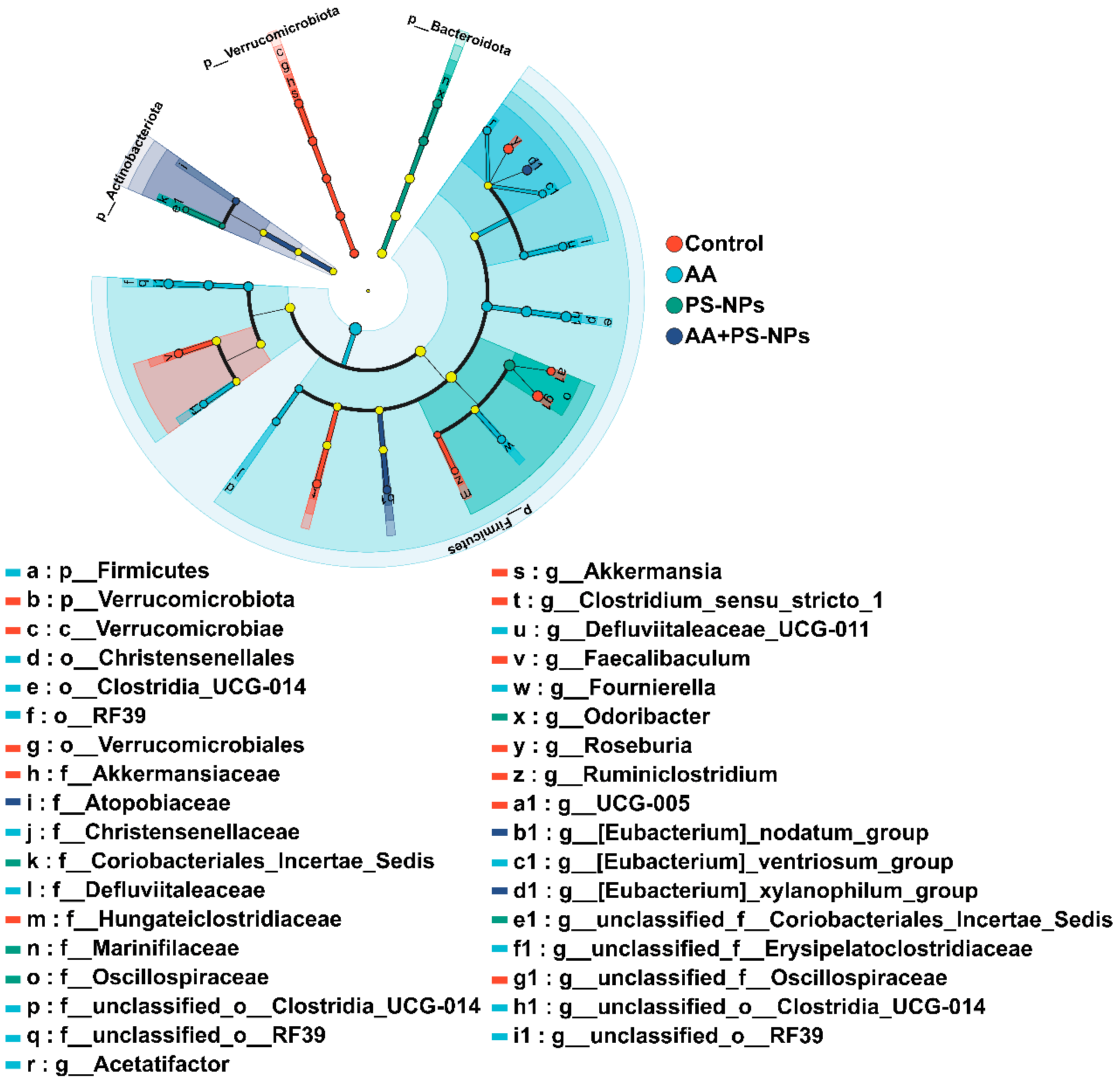

Figure S2 The Cladogram bacterial taxa analyzed by LEfSe (LDA score > 2.5, $p < 0.05$).

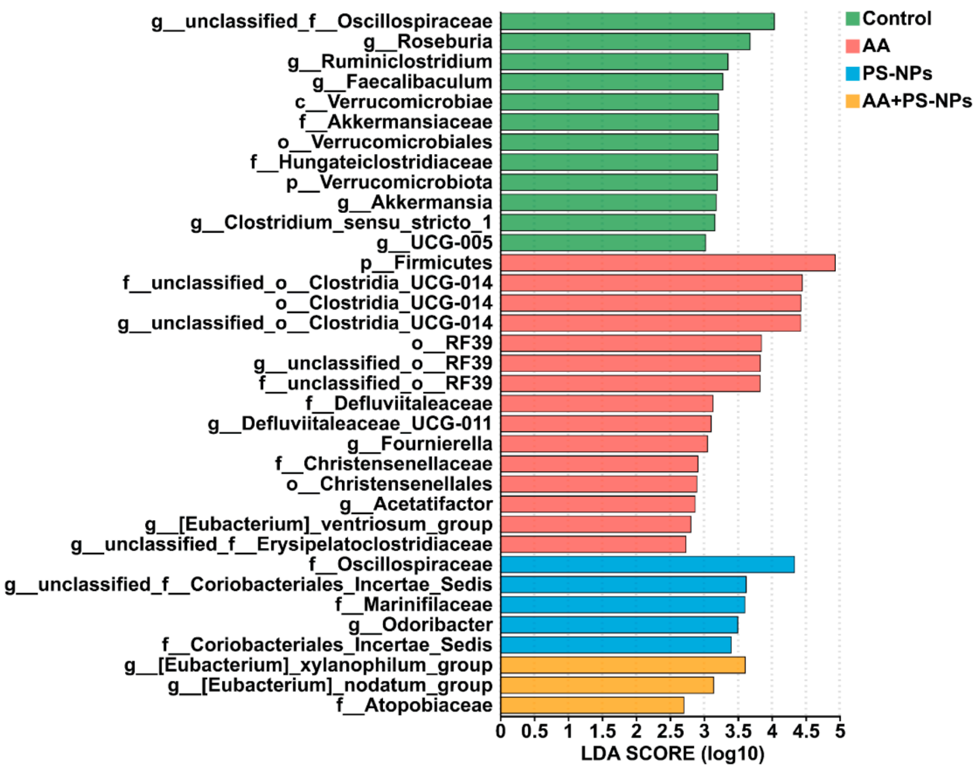

Figure S3 LDA score histogram from the phylum to genus level by LEfSe (LDA score > 2.5, $p < 0.05$).
